# Bioavailability of Polyphenol Liposomes: A Challenge Ahead

**DOI:** 10.3390/pharmaceutics5030457

**Published:** 2013-09-17

**Authors:** Nathalie Mignet, Johanne Seguin, Guy G. Chabot

**Affiliations:** Chemical, Genetic and Imaging Pharmacology Laboratory (CNRS UMR 8151, INSERM U1022), Faculty of Pharmacy, Paris Descartes University, Sorbonne Paris Cité, Chimie-ParisTech, 4 avenue de l’Observatoire, Paris F-75006, France; E-Mail: johanne.seguin@parisdescartes.fr

**Keywords:** polyphenols, flavonoids, liposomes, lipid polyphenol interaction, bioavailability

## Abstract

Dietary polyphenols, including flavonoids, have long been recognized as a source of important molecules involved in the prevention of several diseases, including cancer. However, because of their poor bioavailability, polyphenols remain difficult to be employed clinically. Over the past few years, a renewed interest has been devoted to the use of liposomes as carriers aimed at increasing the bioavailability and, hence, the therapeutic benefits of polyphenols. In this paper, we review the causes of the poor bioavailability of polyphenols and concentrate on their liposomal formulations, which offer a means of improving their pharmacokinetics and pharmacodynamics. The problems linked to their development and their potential therapeutic advantages are reviewed. Future directions for liposomal polyphenol development are suggested.

## 1. Introduction

Natural polyphenols are widely distributed in the vegetal kingdom and are defined as organic chemicals characterized by the presence of at least one phenol unit. Polyphenols are usually divided into several classes, depending on their basic chemical structures, into phenolic acids, stilbenes, lignans and flavonoids. Flavonoids constitute the most important family, with approximately 6000 molecules identified so far. These compounds are secondary plant metabolites involved in important biological processes (germination, UV protection, insecticides) and are also involved in the attraction of pollinating agents via the vivid colors of the anthocyanin pigments found in flowers [1,2,3]. 

Polyphenols have been intensely studied, partly because of a renewed interest in medicinal plants used in traditional medicine [4] and, also, because the so-called Mediterranean diet rich in fruits, vegetables and red wine appears to protect against cardiovascular diseases [5]. Polyphenols have also attracted a particular attention, because of their potential beneficial health properties, as evidenced by several epidemiological studies showing that diets rich in fruits and vegetables are generally associated with a lower cancer incidence [6,7,8] and other diseases, such as inflammatory or cardiovascular pathologies [9].

Humans are exposed daily to polyphenols through their diet composed of green vegetables, onions, fruits (apples, grapes, strawberries, *etc*.), soya bean-derived products (isoflavonoids) and beverages, like coffee, tea, beer and red wine, but the consumption of polyphenols appears to be highly variable between different countries [10,11,12]. However, because the evaluation of the human intake of flavonoids is usually based only on the consumption of a few flavonoids, the actual daily intake of flavonoids is probably superior to the reported estimates, which are in the range of three to 68 mg/day, with a median value of 23 mg/day [13]. Some authors have estimated the daily consumption of polyphenols to be higher, *i.e.*, in the range of 150 to 1000 mg/day [14]. Fruits (mainly apples and strawberries) and vegetables (e.g., potatoes, lettuce, onions) account for about 28% of the daily polyphenol intake, and the total consumption would be over 300 mg per day in France [15]. Because fruits, vegetables, tea, coffee and red wine are all rich in polyphenols, the focus of several research teams is now to identify which polyphenol is responsible for a given pharmacological or preventive effect, and also, efforts are devoted to try to decipher the molecular mechanisms of action.

The chemical properties of polyphenols are difficult to address, as they will depend on the number of phenyl rings in the molecule and the number of hydroxyl groups present on these aromatic cycles (reviewed in [16]). In addition to its chemical structure, the bioavailability of polyphenols contained in a given food will depend on the conditions where the plants have been harvested, food processing and matrix, interaction with other compounds in the food extract and host related factors, like gender, age or health conditions (reviewed in [17,18]).

Despite their putative health promoting properties, the bioavailability of orally administered polyphenols appears insufficient to allow high enough drug concentrations for systemic therapy. Indeed, low water solubility, poor absorption and extensive and rapid metabolism contribute to the low oral bioavailability of polyphenols [19]. These problems can be tackled using different drug delivery approaches in order to improve their bioavailability and, hopefully, their therapeutic efficacy.

**Figure 1:**
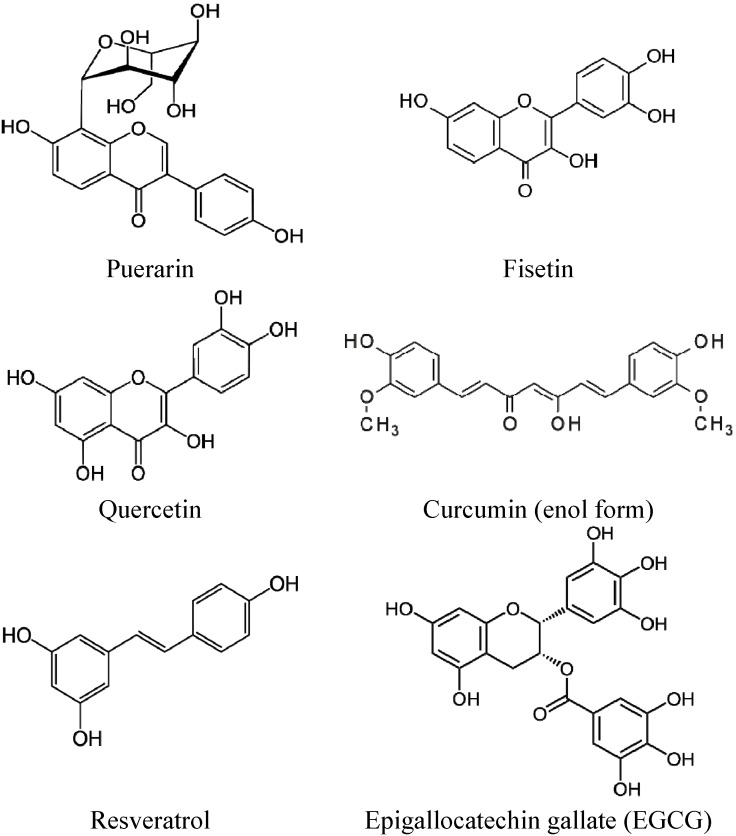
Chemical structures of some polyphenols mentioned in this review.

Several polyphenols have been formulated into various pharmaceutical formulations that could solubilize, stabilize or increase their bioavailability. Examples include the following: pro-drug design [20], formulation with cyclodextrin [21], simple emulsions, self-emulsifying delivery systems, gels, lipid nanocapsules [22], nanoemulsion [23] or liposomes [24,25]. All formulations appear to improve the solubility of the various polyphenols, e.g., puerarin, resveratrol, quercetin, curcumin, fisetin and epigallocatechin gallate (EGCG) (Figure 1). 

Several good review articles have been devoted to the use of nanotechnologies to improve polyphenol delivery [26]. In the present review, we will mainly focus on liposomal formulations of polyphenols.

## 2. Liposomal Formulations of Polyphenols

Liposomes are excellent candidates for drug delivery, because of their biocompatibility, the possibility to fine-tune their physico-chemical properties according to their lipid composition and content and, also, due to the possibility to tailor their surface composition. These vesicles are indeed obtained from naturally occurring phospholipids or from synthetic mimetic lipids surrounding an aqueous core. Liposomes have initially been proposed by Gregoriadis [27,28] and have been the only nanoparticles available on the market, since 2008. Their long-standing commercialization with various classes of molecules, such as doxorubicin, amphotericin B, verteporfin, cytarabine, vincristine or inactivated hepatitis B virus, give the liposomes an advantage over other nanocarriers, because of the technical and efficacy knowledge gathered over the years [29,30]. Several companies have since been launched to optimize the processes and to produce good manufacturing process (GMP) liposome batches [31]. 

Although liposomes are considered to be able to entrap both hydrophilic and lipophilic drugs into their aqueous compartment and their lipidic bilayer, respectively [32], one can easily guess that lipophilic drugs will not be encapsulated with high efficiency without disturbing the membrane bilayer integrity [33]. However, liposomes have been proposed for polyphenol encapsulation. One can wonder whether encapsulating polyphenols into liposomes can result in sufficient liposomal stability. Actually, this feasibility strongly depends on the polyphenol compound itself. As alluded to above, polyphenols are very diverse molecules in which the number of rings and hydroxyl groups will greatly influence the polyphenol solubility in surfactants.

It is also noteworthy that most liposomal forms of polyphenol reported in the literature have not been developed for pharmaceutical purposes, but for the use of their antioxidant properties in food. These studies are critical for the development of polyphenol liposomal forms, as they address the difficult question of polyphenol interaction with membranes.

### 2.1. Lipid-Polyphenol Molecular Interactions

Initial studies showing that flavonoids exhibit a protective antioxidizing effect in aqueous media also showed a protective antioxidizing effect on lipids. It was not clear, then, if this effect was due to a particular positioning of the polyphenol at the surface of the liposomes or if these molecules would rather partition in the lipidic compartment [34]. Epigallocatechin gallate (EGCG) has been shown to interact with the phosphate of dimyristoyl-phosphatidylcholine (DMPC) lipid models using ^31^P NMR [35]. A recent study indicated that resveratrol interacts with the DPPC choline group (CH_3_) in conventional DPPC/cholesterol (Chol) liposomes [36]. Replacing the cholesterol by a cationic cholesterol derivative led to a deeper incorporation of the resveratrol in the lipid bilayer. Resveratrol was shown, in this case, to interact with the methylene group of DPPC within the lipid bilayer by ^1^H NMR studies [36]. An adjustment between the amount of cholesterol into the liposomes and the flavonoid, fisetin, indicated that reducing the amount of cholesterol into liposomes allowed the incorporation of more fisetin. In that study, the lipidic bilayer integrity was checked by electronic transmission microscopy [37]. Incorporating more fisetin altered the lipid bilayer, suggesting that part of the fisetin molecule incorporated within the lipid compartment. 

Solubility studies on resveratrol, fisetin or quercetin showed an increased solubility of these polyphenols in oil, but principally for the most hydrophilic ones (hydrophilic-lipophilic balance (HLB) >10) [23,38,39]. In the studies on lipid nanocapsules [22] and emulsions [23], it appears that quercetin and fisetin distribute, rather, at the oil/tensioactive interface. These compounds can then be qualified as neither fully lipophilic, nor hydrophilic, but, rather, amphiphilic, necessitating hydrophobic interactions, as well as hydrogen bond interactions with the aqueous medium to be encapsulated. The presence of Brij98 located at the surface in the liposome/emulsion blend reduced resveratrol release, thus confirming the need of interaction between polyphenol/hydrophilic components [40]. This is consistent with the work of Uekusa *et al.* [41], who indicated that incorporation of polyphenols into lipid membranes was dependent on their chemical structure. These authors also showed that the partition coefficient on a model membrane correlated well with the incorporation of polyphenol into liposomes. The interaction of bile salt with curcumin embedded in DPPC liposomes also showed an interaction between the OH groups of sodium cholate and curcumin [42].

Although not fully understood, the interactions of polyphenols with bilayers include: (a) the partition of the more non-polar compounds in the hydrophobic bilayer of the membrane; and (b) the formation of hydrogen bonds between the more hydrophilic polyphenol and the polar head groups of lipids and at the membrane interface [43]. The requirement of both types of interactions confirms the potential role liposomes can play in delivering polyphenols more efficiently. This is clearly reminiscent of the behavior of cholesterol, the common component of the lipid bilayer, which is located within the lipid bilayer with its hydroxyl available for hydrogen bonding with the aqueous environment. The consequences of those interactions will obviously influence both the encapsulation efficiency and the release rate of polyphenol.

### 2.2. Influence of the Lipid Charge in the Encapsulation and the Release of Polyphenol

Because polyphenols are mostly uncharged molecules, a possible influence of the surface liposome charge is not expected *a priori*. However, incorporation of cationic cholesterol into the formulation was shown to incorporate trans-resveratrol more deeply into the lipid bilayer [36]. Moreover, using anionic lipids, such as dicetyl phosphate or deoxycholic acid, into egg phosphatidylcholine (EPC)/Chol liposomes increased catechin and EGCG release [44]. Even though the effects of charged lipids on polyphenol encapsulation and release might be indirect, it should be taken into account. The permeability and rigidity of the lipid bilayer is indeed influenced by the charge and structure of the incorporated lipids, which is not trivial for polyphenol encapsulation. Moreover, perhaps more important than hydrogen bonding, electro π-cation interactions have also been proposed [45]. Playing with the chemical interaction between the components of the lipids and the polyphenol functions might help in improving the encapsulation rate of the polyphenol into macromolecular assemblies. As an example, Ke *et al.* [46] showed that the complexation between curcumin and dodecyltrimethylammonium bromide (DTAB) is dependent on the interaction forces, surfactant aggregation state and structural alterations of curcumin. Thus, a better understanding of the forces mediating the interaction between the polyphenol and the surfactants could probably improve the loading of polyphenol into liposomes.

## 3. Biological Effects of Liposomal Polyphenols

Although the encapsulation yield remains a critical issue in liposomal formulations of polyphenols, several reports have clearly indicated that the bioavailability and efficacy of polyphenols are indeed improved by liposomes. Table 1 presents a summary of selected liposomal formulations of various polyphenols that have been reported in the literature. When available, the drug/lipid ratio has been indicated, as well as the formulation process and the biological effect obtained. 

As stated above, polyphenols suffer from low solubility, and their interaction with lipids helps to solubilize these compounds. As indicated in Table 1, the encapsulation ratio is highly variable, even for the same polyphenol, which appears to be due to the formulation technology employed and the lipid composition used (Table 1). Basically, most of the studies cited in this review have used film hydration technology, followed by either sonication with a probe or extrusion to obtain liposomes containing polyphenol (Table 1). This suggests that SUV and MLV have mostly been obtained, although this information is lacking in most of the cited articles. As indicated above, the encapsulation rate should mostly be dependent on the polyphenol structure and the lipid composition, although the formulation seems to have an impact, as well. If one looks closely to the encapsulation rate according to the formulation, a trend of improvement can be extracted from these data. The film hydration technique tends to give a range of 0.5% to 10% encapsulation rate (*w*/*w*), depending on the lipid composition and the polyphenol used. An increased encapsulation seems to be achieved when lyophilizing or freeze-drying the liposomes obtained by the film hydration technique. Hence, Li *et al.* [47] or Yuan *et al.* [24] reached a 10% to 30% encapsulation rate (*w*/*w*) by drying the preformed polyphenol/liposomes, suggesting that forcing the interaction between the polyphenol and liposomes could be advantageous. A combination of techniques starting from a film that was hydrated in the presence of ethanol, then sonicated in the presence of additional surfactants and, finally, freeze-dried also led to an improved encapsulation yield [48]. Finally, the proliposomes strategy, initially described by Song *et al.* [49], was applied to obtain liposomes for oral delivery with a 20% encapsulation rate of silymarin [50].

In addition to solubilization improvement, another important advantage of formulating polyphenol with liposomes is the increased chemical stability [51], which could lead to either a prolonged efficacy [52] or could induce an effect that could not be evidenced with the unformulated compound [53]. Moreover, the improved drug solubility within liposomes allows injecting of the active polyphenol into non-organic solvents, which contributes toward decreasing systemic toxicity and may level up the maximum tolerated dose, allowing a higher dose of polyphenol to be administered *in vivo*.

**Table 1:** Examples of liposomal forms developed for polyphenol biological studies.

Polyphenol	Formulation	% (*w*/*w*) ^1^	Effect of the formulation ^2^	References
Catechin	Epikuron 200/Chol/Tween 80/ethanol 6/1/80/13, film hydration	0.3	Increased bioavailability and cerebral distribution	[54]
Curcumin	SPC; film Hydration, extrusion, MLV	3	Prolonged antioxidant protective effect	[52]
Curcumin	DMPC or DMPC/DMPG lyophilisate	10 to 25	Similar efficacy *in vitro*. Antiangiogenic effect and tumor growth reduction *in vivo*	[47,55]
Curcumin/resveratrol	DMPC, lyophilisate	20	Improved bioavailability and reduction of prostate cancer incidence	[56]
Dehydro-silymarin	SPC/Chol/IPM/sodium cholate 1.5/0.3/1/1 film + freeze-drying	25	Increased oral bioavailability	[48]
EGCG Catechin	EPC/Chol/DA 4/1/0.25 film hydration + sonication or extrusion	20	Protection from degradation; Increased carcinoma cell death at lower concentrations	[53]
Fisetin	DOPC/DOPC/DODA-PEG_2000_^/^Fis 8/1.3/0.4/0.3 film hydration + extrusion, MLV	18	Increased bioavailability and antitumor efficacy	[25,37]
Quercetin	PE/Chol/DPC/QC 7/1/1/1 film hydration + sonication	10	Antioxidative effect with the formulation (i.v.)	[57]
Quercetin	Lecithin/Chol/ PEG 4000film hydration + lyophilization, SUV	30	Increased solubility, bioavailability and antitumor efficacy *in vivo* (i.v.)	[24]
Resveratrol	DPPC/DSPE PEG2000/Chol 1.85/0.15/1 film Hydration + extrusion	0.1–5	Improved solubility and chemical stability	[51]
Resveratrol	P90G/DCP/Chol sonication + extrusion	1.5	Prolonged efficacy and improved protection from UV B	[58]
Silymarin	Lecithin/Chol/stearyl amine/Tween 20 9/1/1/0.5 film hydration		Increased stability, bioavailability and liver protection	[59,60]
Silymarin	Mannitol, phospholipids proliposomes	20	Improved oral bioavailability	[50]

^1^ percent of polyphenol/lipids; ^2^ effect as compared to the free drug. Abbreviations: EPC, egg phosphatidylcholine; SPC, soybean phosphatidylcholine; DMPC, dimyristoyl-phosphatidylcholine; DPPC, dipalmytoyl-phosphatidylcholine; DOPC, dioleoyl-phosphatidylcholine; DMPG, dimyristoyl-phosphatidylglycerol; DODA-PEG, dioctadecylcarbamoylmethoxyacetylamino)acetic acid(methoxy)-polyethylene glycol; DSPE-PEG, distearoyl-phosphatidylethanolamine-polyethylene glycol; Chol: cholesterol; DC-Chol, dimethylaminomethane-carbamoyl-cholesterol; DA, deoxycholic acid; QC, quercetin; DCP, dicetyl phosphate; P90G, phospholipon 90G; IPM: isopropyl myristate.

*In vitro* studies with polyphenol inserted into liposomes usually show an improved or a similar efficacy compared to the unformulated polyphenol [37,47]. For instance, Tonnesen *et al.* showed a 20-fold increase of curcumin concentration in red blood cells when curcumin was formulated into liposomes as compared to being diluted in DMSO [47]. Encapsulation of resveratrol allowed more efficacy in terms of protection against UV B [58]. The improved solubility of quercetin with liposomes induced an increase bioactivity, which was explained by a longer exposure of the cells to the active substance [61]. For other groups, a similar activity of polyphenol formulated into liposomes was noticed. This is the case of the cytotoxicity obtained with the liposomal form of trans-resveratrol [36] or the liposomal form of fisetin [37].

**Figure 2:**
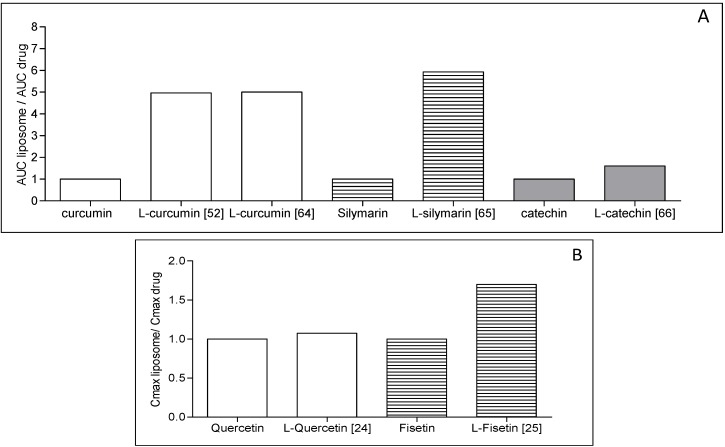
Biological interest of formulating polyphenol into liposomes. The histograms are based on the references indicated in the figure. Area under the curve (AUC) for the free polyphenol was fixed at level 1; the level given for the liposomal form of polyphenol corresponds to the AUC increase factor as compared to the free polyphenol (**A**) for orally administered liposomes; (**B**) for intravenously administered liposomes (*cf.* [24,25,50,54,62,63]).

Concerning the *in vivo* situation, the advantage of the polyphenol liposomal formulation has been shown in several studies. Oral bioavailability is often improved with liposomes, as shown with catechin, dehydrosilymarin, quercetin, puerarin and silymarin (*cf.* Table 1). We tried to evaluate in this review the benefit of incorporating polyphenol into liposomes in terms of the concentration found in the plasma, as compared to free polyphenol. Based on the area under the curve (AUC) data given in the literature, we artificially fixed the level of free polyphenol at one and implemented the level of the polyphenol under its liposomal form found in the plasma (Figure 2). For example, liposomal catechin administered orally was found to increase by a factor of 1.6 area under the curve (AUC) of the catechin in the plasma as compared to the free drug. Takahashi *et al.* [62] observed an enhancement of a factor of five when curcumin was administered orally into liposomes, as compared to curcumin alone or to a mixture of curcumin and lipids used in the formulation (Figure 2A). Basically, for the free polyphenol described, a factor of 1.6 to five was obtained in favor of the liposomal form. 

Systemic administration with liposomes by parenteral routes (e.g., intravenous, intraperitoneal) also led to improved bioavailability in several studies [24,25,57]. In the case of fisetin liposomal encapsulation, an increased fisetin concentration and a lower elimination in the plasma of mice was measured (Figure 2B). 

What is particularly interesting is that overcoming the low bioavailability of polyphenol translated into an increased therapeutic effect *in vivo*, as mentioned in Coimbra *et al.* [51]. As an example, systemic injection of fisetin and quercetin under their liposomal forms led to tumor growth delay [24,25]. Liposomal curcumin suppressed the growth of both BxPC3 and MiaPaCa_2_ tumors in murine models after i.v. injection. The authors showed that this effect was due to an inhibition of angiogenesis as the expression of CD31, VEGF and IL-8 decreased, while the empty liposomes had no effect [47].

Liposome improvement in bioavailability appears to be due to several factors. A prolongation in residence time is often observed with the liposomal formulation compared to the non-formulated polyphenol [24,25]. This prolonged residence time probably results from an increased stability of the polyphenol, as it was observed that quercetin in liposome was active at doses where free quercetin is not active [57]. The observed liver accumulation of the liposomal form of polyphenol could eventually act as a slow releasing reservoir that could prolong the residence time of the polyphenol in the blood. This prolonged half-life contributes to enhanced area under the plasma *versus* the time curve, which is linked to the better effectiveness of a given polyphenol. In addition to prolonged residence time in the blood, the better intracellular passage of the liposomal polyphenol into the cell probably plays a major role to elicit a better therapeutic effect in cancer and other diseases responding to polyphenol therapy.

## 4. Liposome Development Issues

Liposomes are the nanoparticles on which we have the most experience, due to products having already been commercialized. Due to this vast experience on liposomes, one would have expected more liposomal formulations to be put on the market, in correspondence to the vast literature on drug delivery with liposomes. Liposomes have tremendous advantages, like increased drug stability and bioavailability. Furthermore, because of their size, liposomes can flow easily in blood circulation and sustain distortion, such as red blood cells. In addition, liposomes are retained in inflamed tissues, taking advantage of discontinuous endothelia. Besides their advantages, one of the main limitations of liposomes is their stability upon storage and rapid elimination after administration. 

Concerning the storage issue, encapsulation of polyphenols is fortunately taking advantage of the inherent polyphenol antioxidant properties, which can be highly beneficial for the lack of stability of liposomes, due to the oxidation of unsaturated lipid. Atrooz *et al.* showed that polyphenols can reduce lipid oxidation and aggregation and improve their storage stability [64]. Therefore, encapsulation of polyphenol into liposomes can stabilize the polyphenol itself, but could also favor liposome stability and storage.

For rapid blood elimination, liposome surface modifications have been proposed. PEGylation of liposomes has indeed markedly improved their half-life, from a few minutes to several hours, by reducing their interaction with blood proteins [65]. Other types of liposomal coverings have also been proposed [66]. A higher dose of liposomes can also increase the residence time of liposomes, due to saturation of blood protein interactions [67].

In principle, liposomes are highly flexible, meaning that lipid composition can be adapted to the drug, and the surface can be modified to achieve long-term circulation or targeting properties. Lipid composition should indeed be tuned to increase the encapsulation of poorly soluble drugs [68]. An increased permeability of the lipid bilayer should, in principle, be beneficial, as shown for catechin [66]. The relation between the lipid geometry in the liposomes and the encapsulation of insoluble drugs has been studied [69]. In addition, the polyphenol geometry should be taken into account to evaluate the feasibility of its effective insertion into lipid bilayers. As of today, there is no status quo allowing the generalization of lipid composition and encapsulation. Research must still focus on lipid composition and the surface modification of liposomes, in order to enhance the incorporation of insoluble drugs into liposomes and, also, to improve liposome targeting to well-defined targets. 

## 5. Concluding Remarks

Polyphenols can be regarded as compounds possessing clear-cut pharmacological activities in a variety of diseases, including cancer prevention and treatment, as was demonstrated in several *in vitro* and *in vivo* preclinical systems. Because of the poor oral bioavailability of polyphenols in their aglycone forms, much work needs to be accomplished to overcome this serious problem before the use of these agents could be recommended as therapeutics in humans. 

As shown in this review, liposomes can increase polyphenol solubility and stability, which translate into improved bioavailability and therapeutic benefit. Although these promising results have not been yet translated to the clinic, it should be noted that the research on this topic is relatively recent, because most of the articles found on this topic were published within the last ten years. Liposomes are already in the clinic and can be produced at a large scale. Although the encapsulation rate still needs to be improved for polyphenols, the increased solubility and efficiency resulting from this encapsulation could lead to potentially efficacious clinical products.

Based on the growing interest of the scientific community in the study of natural product use in medicine, it seems likely that, in addition to liposomes, the next decade will see the emergence and maturation of new, improved polyphenol pharmaceutical formulations, e.g., nanoparticles, microemulsions and polymeric implantable devices [70]. Chemoprevention, in order to keep cancer from forming, growing or coming back, is one of the goals that could be eventually achieved with certain polyphenols. For example, resveratrol microparticles have already undergone preclinical investigation for their cancer chemopreventive properties and went to phase I clinical trial. The mean plasma level was 3.6 fold higher compared to non-micronized resveratrol, and increased apoptosis of hepatic cancer cells was also observed, hinting that this formulation could offer a therapeutic advantage over non-micronized resveratrol [71]. Curcumin formulated as an oral capsule (containing also green tea, *Polygonum cuspidatum* and soybean extracts) is also undergoing phase I trial in the prevention of colon cancer [72]. 

The design of prodrugs that could be better absorbed is also under way [73]. Modification of the parent polyphenol, e.g., via permethylation, could also be helpful to increase their metabolic stability and to prolong their residence time *in vivo* [74]. Structural modification of resveratrol has been reported to markedly improve its half-life and therapeutic efficacy [75]. It is hoped that these formulations will improve the clinical effectiveness of polyphenols and contribute to their use in the prevention and curing of major human illnesses.

## Abbreviations

Chol: cholesterol
DA: deoxycholic acid
DC-Chol: dicarbamate-cholesterol
DMPC: dimyristoyl-phosphatidylcholine
DODA-PEG: dioctadecylcarbamoylmethoxyacetylamino)acetic acid(methoxy)-polyethylene glycol
DOPC: dioleoyl-phosphatidylcholine
DPPC: dipalmitoyl-phosphatidylcholine
EGCG: epigallocatechin gallate
EPC: egg phosphatidylcholine
HLB: hydrophilic-lipophilic balance
QC: quercetin, NMR, Nuclear Magnetic Resonance
SUV: Small Unilamellar Vesicle
MLV: Multilamellar Vesicle
DMSO: dimethylsulfoxide
CD-31: Cluster of Differentiation 31
VEGF: Anti-vascular endothelial growth factor
IL-8: Interleukin-8
